# Supplementary material for: Modeling glioblastoma heterogeneity as a dynamic network of cell states
Source: Mol Syst Biol. 2021 Sep 16;17(9):e10105. doi: 10.15252/msb.202010105 (PMC8444284; doi:10.15252/msb.202010105)
Supplement: Supplementary file 5 — Source Data for Figure 3 [file MSB-17-e10105-s001.zip › Figure3A_sourcedata/GSEA_3065/hallmarks_state2.GseaPreranked.1623416269049/HALLMARK_KRAS_SIGNALING_UP.html]

Details for gene set HALLMARK\_KRAS\_SIGNALING\_UP[GSEA]

|  || Dataset | state2 |
| Phenotype | NoPhenotypeAvailable |
| Upregulated in class | na\_neg |
| GeneSet | HALLMARK\_KRAS\_SIGNALING\_UP |
| Enrichment Score (ES) | -0.5285713 |
| Normalized Enrichment Score (NES) | -1.9954016 |
| Nominal p-value | 0.0 |
| FDR q-value | 0.0 |
| FWER p-Value | 0.0 |
Table: GSEA Results Summary

  

Fig 1: Enrichment plot: HALLMARK\_KRAS\_SIGNALING\_UP      
 Profile of the Running ES Score & Positions of GeneSet Members on the Rank Ordered List

  

| PROBE | GENE SYMBOL | GENE\_TITLE | RANK IN GENE LIST | RANK METRIC SCORE | RUNNING ES | CORE ENRICHMENT || 1 | CCND2 |  |  | 227 | 0.263 | 0.0109 | No |
| 2 | TRIB2 |  |  | 636 | 0.158 | -0.0117 | No |
| 3 | GNG11 |  |  | 698 | 0.151 | 0.0019 | No |
| 4 | SPRY2 |  |  | 870 | 0.131 | 0.0011 | No |
| 5 | DUSP6 |  |  | 1345 | 0.095 | -0.0369 | No |
| 6 | SOX9 |  |  | 1466 | 0.088 | -0.0380 | No |
| 7 | PRELID3B |  |  | 1506 | 0.086 | -0.0307 | No |
| 8 | GYPC |  |  | 1659 | 0.079 | -0.0364 | No |
| 9 | ETV4 |  |  | 1840 | 0.071 | -0.0462 | No |
| 10 | ETV5 |  |  | 1843 | 0.071 | -0.0370 | No |
| 11 | CA2 |  |  | 2111 | 0.061 | -0.0574 | No |
| 12 | MAP3K1 |  |  | 2140 | 0.060 | -0.0524 | No |
| 13 | ZNF639 |  |  | 2195 | 0.058 | -0.0505 | No |
| 14 | STRN |  |  | 2519 | 0.047 | -0.0787 | No |
| 15 | YRDC |  |  | 2559 | 0.046 | -0.0768 | No |
| 16 | CBL |  |  | 2642 | 0.043 | -0.0798 | No |
| 17 | FBXO4 |  |  | 2891 | 0.037 | -0.1014 | No |
| 18 | ETS1 |  |  | 2998 | 0.034 | -0.1082 | No |
| 19 | MMD |  |  | 3023 | 0.033 | -0.1064 | No |
| 20 | NIN |  |  | 3079 | 0.032 | -0.1080 | No |
| 21 | ETV1 |  |  | 3908 | 0.017 | -0.1942 | No |
| 22 | PSMB8 |  |  | 3949 | 0.016 | -0.1963 | No |
| 23 | BPGM |  |  | 4537 | 0.007 | -0.2582 | No |
| 24 | WDR33 |  |  | 4575 | 0.006 | -0.2613 | No |
| 25 | BTBD3 |  |  | 4597 | 0.006 | -0.2628 | No |
| 26 | PCP4 |  |  | 4628 | 0.005 | -0.2653 | No |
| 27 | PEG3 |  |  | 4783 | 0.003 | -0.2813 | No |
| 28 | AMMECR1 |  |  | 4983 | 0.000 | -0.3026 | No |
| 29 | USP12 |  |  | 5029 | -0.000 | -0.3074 | No |
| 30 | SCN1B |  |  | 5213 | -0.004 | -0.3264 | No |
| 31 | MTMR10 |  |  | 5281 | -0.005 | -0.3330 | No |
| 32 | VWA5A |  |  | 5352 | -0.006 | -0.3397 | No |
| 33 | ERO1A |  |  | 5380 | -0.006 | -0.3417 | No |
| 34 | KIF5C |  |  | 5587 | -0.010 | -0.3624 | No |
| 35 | RABGAP1L |  |  | 5747 | -0.012 | -0.3778 | No |
| 36 | ANKH |  |  | 5757 | -0.012 | -0.3771 | No |
| 37 | ZNF277 |  |  | 5852 | -0.014 | -0.3853 | No |
| 38 | AKT2 |  |  | 6104 | -0.018 | -0.4097 | No |
| 39 | MMP11 |  |  | 6202 | -0.020 | -0.4174 | No |
| 40 | PTCD2 |  |  | 6355 | -0.023 | -0.4306 | No |
| 41 | TSPAN7 |  |  | 6363 | -0.023 | -0.4283 | No |
| 42 | ATG10 |  |  | 6374 | -0.023 | -0.4264 | No |
| 43 | AKAP12 |  |  | 6471 | -0.025 | -0.4333 | No |
| 44 | GADD45G |  |  | 6483 | -0.025 | -0.4311 | No |
| 45 | TOR1AIP2 |  |  | 6492 | -0.025 | -0.4286 | No |
| 46 | CCSER2 |  |  | 6522 | -0.026 | -0.4283 | No |
| 47 | G0S2 |  |  | 6695 | -0.029 | -0.4428 | No |
| 48 | SEMA3B |  |  | 6759 | -0.031 | -0.4454 | No |
| 49 | PLAUR |  |  | 6906 | -0.034 | -0.4564 | No |
| 50 | PPP1R15A |  |  | 7004 | -0.037 | -0.4619 | No |
| 51 | DNMBP |  |  | 7277 | -0.044 | -0.4851 | No |
| 52 | SDCCAG8 |  |  | 7425 | -0.048 | -0.4944 | No |
| 53 | RGS16 |  |  | 7474 | -0.050 | -0.4929 | No |
| 54 | EVI5 |  |  | 7574 | -0.052 | -0.4965 | No |
| 55 | SATB1 |  |  | 7741 | -0.058 | -0.5065 | No |
| 56 | APOD |  |  | 7757 | -0.058 | -0.5003 | No |
| 57 | PTBP2 |  |  | 7861 | -0.062 | -0.5031 | No |
| 58 | GLRX |  |  | 8100 | -0.071 | -0.5191 | Yes |
| 59 | PLAU |  |  | 8131 | -0.073 | -0.5125 | Yes |
| 60 | CROT |  |  | 8253 | -0.080 | -0.5147 | Yes |
| 61 | F2RL1 |  |  | 8306 | -0.083 | -0.5092 | Yes |
| 62 | LY96 |  |  | 8332 | -0.085 | -0.5005 | Yes |
| 63 | ADAM17 |  |  | 8339 | -0.085 | -0.4898 | Yes |
| 64 | AVL9 |  |  | 8374 | -0.087 | -0.4818 | Yes |
| 65 | FUCA1 |  |  | 8399 | -0.089 | -0.4725 | Yes |
| 66 | PRRX1 |  |  | 8427 | -0.091 | -0.4633 | Yes |
| 67 | CBR4 |  |  | 8669 | -0.109 | -0.4746 | Yes |
| 68 | PRDM1 |  |  | 8693 | -0.110 | -0.4623 | Yes |
| 69 | TSPAN13 |  |  | 8713 | -0.112 | -0.4494 | Yes |
| 70 | ID2 |  |  | 8781 | -0.120 | -0.4406 | Yes |
| 71 | CPE |  |  | 8816 | -0.125 | -0.4276 | Yes |
| 72 | HDAC9 |  |  | 8833 | -0.127 | -0.4123 | Yes |
| 73 | TMEM158 |  |  | 8846 | -0.128 | -0.3965 | Yes |
| 74 | KCNN4 |  |  | 8856 | -0.130 | -0.3801 | Yes |
| 75 | RBM4 |  |  | 8941 | -0.140 | -0.3705 | Yes |
| 76 | GPRC5B |  |  | 8956 | -0.142 | -0.3530 | Yes |
| 77 | TFPI |  |  | 9060 | -0.159 | -0.3427 | Yes |
| 78 | PLAT |  |  | 9145 | -0.176 | -0.3281 | Yes |
| 79 | EMP1 |  |  | 9156 | -0.179 | -0.3053 | Yes |
| 80 | GPNMB |  |  | 9290 | -0.228 | -0.2891 | Yes |
| 81 | PCSK1N |  |  | 9321 | -0.247 | -0.2594 | Yes |
| 82 | CXCR4 |  |  | 9328 | -0.251 | -0.2265 | Yes |
| 83 | SPARCL1 |  |  | 9360 | -0.288 | -0.1914 | Yes |
| 84 | SPP1 |  |  | 9415 | -0.436 | -0.1390 | Yes |
| 85 | IGFBP3 |  |  | 9424 | -0.502 | -0.0729 | Yes |
| 86 | DCBLD2 |  |  | 9431 | -0.558 | 0.0010 | Yes |
Table: GSEA details [plain text format]

  

Fig 2: HALLMARK\_KRAS\_SIGNALING\_UP: Random ES distribution      
 Gene set null distribution of ES for **HALLMARK\_KRAS\_SIGNALING\_UP**
